# Differential Expression and Correlation Analysis of Global Transcriptome for Hemorrhagic Transformation After Acute Ischemic Stroke

**DOI:** 10.3389/fnins.2022.889689

**Published:** 2022-06-08

**Authors:** Rongrong Han, Peng Zhang, Hongfang Li, Yun Chen, Yongnan Hao, Qiang Guo, Aimei Zhang, Daojing Li

**Affiliations:** ^1^Department of Clinical Medicine, Jining Medical University, Jining, China; ^2^Department of Neurology, Affiliated Hospital of Jining Medical University, Jining, China; ^3^Department of Emergency Stroke, Affiliated Hospital of Jining Medical University, Jining, China

**Keywords:** acute ischemic stroke, hemorrhagic transformation, transcriptome sequencing, gene ontology, pathway analysis

## Abstract

In order to explore the epigenetic characteristics of hemorrhagic transformation (HT) after acute ischemic stroke, we used transcriptome sequencing technology to analyze the global transcriptome expression profile of patients with and without HT after acute ischemic stroke and to study the differential expression of messenger RNA (mRNA), long noncoding RNA (lncRNA), circular RNA (circRNA) and mircoRNA (miRNA) between the two groups. To further explore the role of differentially expressed genes in HT, we annotated the function of differentially expressed genes by using gene ontology (GO) and pathway analysis on the results and showed that there were 1,051 differential expressions of lncRNAs, 2,575 differential expressions of mRNAs, 447 differential expressions of circRNAs and 47 miRNAs in patients with HT compared with non-HT patients. Pathway analysis showed that ubiquitin-mediated proteolysis, MAPK signal pathway, axon guidance, HIF-1 signal pathway, NOD-like receptor signal pathway, beta-alanine metabolism, Wnt signal pathway, sphingolipid signal pathway, neuroactive ligand-receptor interaction, and intestinal immune network used in IgA production play an important role in HT. Terms such as iron homeostasis, defense response, immune system process, DNA conformational change, production of transforming growth factor beta-2, and oxidoreductase activity were enriched in the gene list, suggesting a potential correlation with HT. A total of 261 lncRNA-miRNA relationship pairs and 21 circRNA-miRNA relationship pairs were obtained; additionally, 5 circRNAs and 13 lncRNAs were screened, which can be used as competing endogenous RNA (ceRNA) to compete with miRNA in the co-expression network. Co-expression network analysis shows that these differentially expressed circRNA and lncRNA may play a vital role in HT and provide valuable information for new biomarkers or therapeutic targets.

## Introduction

Stroke is a leading cause of mortality and morbidity, of which acute ischemic stroke accounts for about 80%, the key therapy is to restore blood flow and save the ischemic penumbra as soon as possible (Miller et al., 2017). Hemorrhagic transformation (HT) is a spontaneous or thrombolytic complication of acute ischemic stroke and is defined as bleeding caused by re-perfusion of the blood flow in the ischemic area (Alberts, 2017). HT usually occurs during an uncertain period within a few hours or even weeks after stroke and can be classified according to the deterioration of imaging features and clinical symptoms. The common classification criteria were suggested from the European Cooperative Acute Stroke Study I (ECASS I). In this study, bleeding events were divided into the following categories according to clinical and computerized tomography criteria: small petechial hemorrhagic infarction (HI1), confluent petechial hemorrhagic infarction (HI2), small parenchymal hemorrhage (PH1, <30% of infarct, mild mass effect), and large parenchymal hemorrhage (PH2, >30% of infarct, marked mass effect) (Diedler et al., 2010). The National Institute of Neurological Diseases and Stroke (NINDS) divides HT into asymptomatic HT and symptomatic HT (sHT) by brain computed tomography (CT) or magnetic resonance imaging (MRI), which is characterized by clinical deterioration and imaging evidence (Khatri et al., 2007). The clinical classification of HT is as follows: asymptomatic HT, although HTs, there is no clinical deterioration in the National Institutes of Health Stroke Scale (NIHSS) score, mild symptom HT (NIHSS score increased by 1–3 points), and severe symptom HT (Lee et al., 2019). HT is known to be associated with neurological deterioration in at least 20% of patients, which can increase morbidity and mortality (de Andrade et al., 2020). In clinical work, accurate prediction and intervention of HT will show the great significance. Related studies have found a variety of pathophysiological mechanisms involved in the HT, such as neuroinflammation, neurovascular unit injury, blood-brain barrier destruction, and vascular remodeling (Yaghi et al., 2017). However, the epigenetic characteristics of HT have not been fully understood.

Long non-coding RNA (lncRNA), circular RNA (circRNA) and microRNA (miRNA) are three kinds of non-coding RNA (ncRNA) that could modulate many cellular functions. Traditionally, ncRNA refers to not have the ability to translate into proteins. However, current studies have confirmed potential translational activities of some lncRNAs and circRNAs to generate several polypeptides which possess biological functions (Anderson et al., 2015; Matsumoto et al., 2017; Kristensen et al., 2019). lncRNA and circRNA can be used as competing endogenous RNA (ceRNA) to weaken the inhibition of microRNA (miRNA) on targeting messenger RNA (mRNA) (Roberts et al., 2014). LncRNA is a class of RNA molecules whose transcripts are longer than 200 nucleotides, including five different subtypes: sense lncRNA, antisense lncRNA, bidirectional lncRNA, intergenic lncRNA, and intronic lncRNA (Braga et al., 2020). It is reported that lncRNA regulates gene expression through a variety of mechanisms, including regulation of transcription factor activity, splicing mechanism, transcription enhancer, increasing the stability of mRNA, as a structural component of protein complex assembly, and as molecular bait for miRNA (Anfossi et al., 2018; Yao et al., 2019). Studies have shown that lncRNA can affect the function of nerve cells, including neurons, microglia, astrocytes, and oligodendrocytes (Shi et al., 2018). CircRNA is a new type of endogenous RNA formed by covalently closed cyclization, which can interact with RNA-binding proteins to regulate stability and gene transcription of mRNA (Zhang et al., 2017a). Their molecular functions are also varied, including acting as miRNA sponges, regulators of transcription and splicing, ribosomal RNA processing, and aptamers for protein-protein interactions (Hsiao et al., 2017). It is reported that circRNA also plays a key role in neurological diseases, including Alzheimer's disease, ischemic stroke, and cerebral ischemia-reperfusion injury (Qu et al., 2017; Yang et al., 2018a).

The destruction of blood-brain barrier is considered to be an important mechanism of HT. Recent studies showed that ncRNA, which is highly expressed in cerebral microvascular endothelial cells, is considered to be a potential mediator affecting the permeability of blood-brain barrier. Some studies have found that high levels of miR-155 in brain microvascular endothelial cells can increase the permeability of the blood-brain barrier by targeting regulation of Mfsd2a (Awad et al., 2018). miR-182 aggravates the destruction of the blood-brain barrier through the mTOR/FOXO1 pathway (Zhang et al., 2020). LncRNA Snhg3 contributes to dysfunction of cerebral microvascular cells and increases the permeability of BBB by activating the TWEAK/Fn14/STAT3 pathway after cerebral hemorrhage of a rodent model (Zhang et al., 2019). In an oxygen-glucose deprivation model of cerebral microvessel endothelial cells, the levels of lncRNA-MALAT1 and miR-145 were up-regulated, while lncRNA-MALAT1 enhanced the expressions of VEGF-A and ANGPT2 by targeting miR-145 which may affect the integrity of the blood-brain barrier. VEGF-A significantly aggravates the damage of blood-brain barrier after stroke by up-regulating the expression of LOC102540519 and HOXC13 (Wu L. et al., 2018; Ren et al., 2019). CircRNA HECW2 inhibits the expression of miR-30d and promotes the production of ATG5 through Notch/Notch1 pathway, thus aggravates the interstitial transformation of endothelial cells and causes the destruction of BBB (Yang et al., 2018b). Upregulation of circ-FoxO3 and autophagic flux were detected in brain microvessel endothelial cells in patients with hemorrhagic transformation and in mice models with middle cerebral artery occlusion/reperfusion. *In vivo* and *in vitro* studies indicated that circ-FoxO3 alleviated BBB damage principally by autophagy activation via mTORC1 inhibition (Yang et al., 2022). These findings suggest that non-coding RNA may have important regulatory potential in the occurrence of hemorrhagic transformation after stroke.

A comprehensive understanding of the epigenetics and molecular disorders of HT is crucial for early diagnosis, appropriate treatment, and improving the prognosis of patients. Therefore, in this study, transcriptome sequencing technique was used to explore the differentially expressed mRNA, lncRNA, circRNA and miRNA between HT group and non-HT group, and to reveal the complex interactions between their transcripts, to provide useful information for further understanding its mechanism and exploring potential targets for treatment and prognosis.

## Materials and Methods

### Patients and Sample Collection

In this research, we selected 6 patients who were diagnosed as acute ischemic stroke with large artery occlusion and underwent interventional thrombectomy in the Department of Emergency Stroke, Affiliated Hospital of Jining Medical University from October 2020 to June 2021. Inclusion criteria were the following: clinical symptoms, signs, and cranial MRI+MRA examination confirmed acute ischemic stroke with large vessel occlusion; in line with the consensus of Chinese experts on endovascular treatment of acute ischemic stroke with large vessel occlusion (revised in 2019); acute attack, the time from onset to visit is less than 24 hours, which accords with the condition of endovascular treatment; be treated with mechanical thrombectomy; brain CT examination was performed 24 h after thrombectomy. Exclusion criteria were the following: (1) those with a previous history of cerebral hemorrhage; (2) complicated with functional failure of heart, liver, lung, kidney, and other important organs; (3) vasculitis and vascular malformation; (4) complicated with coagulation dysfunction or hemorrhagic disease; (5) the clinical data collection was incomplete. This study has been approved by the ethics committee of the Affiliated Hospital of Jining Medical University. The informed consent and authorized commission are signed by the patient or the patient's authorized representative. The patients were divided into groups according to the results of CT re-examination; Once CT result indicated that transformation of intracranial hemorrhage (cerebral parenchyma hemorrhage), the patient was divided into hemorrhage group (group H or HT), and CT showed that no transformation of intracranial hemorrhage was divided into a non-hemorrhage group (group C or non-HT). There were 3 cases in each group.

Before blood collection, the patients were kept in a fasting state for 12 h. Venous blood 5 mL was taken from EDTA anticoagulant tube to strictly avoid hemolytic samples. 2,000 r/min, 5 min, plasma was removed and diluted with the same volume of PBS. Human peripheral blood mononuclear cells (PBMC) were isolated by the Ficoll-Hypaque gradient method.

### RNA Extraction

For the extraction of RNA, we used TRIzol reagent according to the requirements of the manufacturer and applied the separated PBMC to 1 ml's TRIzol reagent. After mixing fully with the reagent, a single RNA sample was stored in the refrigerator at −80 °C for further use. RNA integrity was assessed with Agilent 2100 Bioanalyzer (Agilent Technologies, Santa Clara, CA, USA) and RNA concentration was measured by Qubit RNA Assay Kit in Qubit Fluorometer (Invitrogen, CA, USA). Total RNA samples that meet the following requirements were used in subsequent experiments: RNA integrity number (RIN) ≥7.0 and a 28S:18S ratio ≥1.5.

### Library Construction Transcriptome Sequencing

Two libraries were constructed in the study. For the Strand-Specific library, 1 μg RNA per sample was used. In brief, The Ribo-Zero™ Magnetic Kit (Epicentre) was used to remove rRNA from the total RNA. The NEBNext Ultra RNA Library Prep Kit for Illumina (NEB, USA) was used to construct the libraries for sequencing following manufacturer's instructions. The RNA was fragmented into pieces of ~200 base pair in length in NEBNext First Strand Synthesis Reaction Buffer (5X). The first-strand cDNA was synthesized from the RNA fragments by reverse transcriptase and random hexamer primers, and then the second-strand cDNA was synthesized in Second Strand Synthesis Reaction Buffer with dUTP Mix (10x). The end of the cDNA fragment was subjected to an end repair process that included the addition of a single “A” base, followed by ligation of the adapters. Products were purified and enriched by polymerase chain reaction (PCR) to amplify the library DNA. The final libraries were qualified by Agilent 2100 and quantified using KAPA Library Quantification kit (KAPA Biosystems, South Africa). Finally, the libraries were subjected to paired-end sequencing with pair end 150-base pair reading length on an Illumina NovaSeq sequencer (Illumina).

For the smallRNA library, 3ug total RNA per sample was used as input material. Sequencing libraries were generated using NEB Next®Multiplex Small RNA Library Prep Set for Illumina® (NEB, USA.). Briefly, NEB 3′ SR Adaptor was directly, and specifically ligated to 3′ end of miRNA, siRNA and piRNA. After the 3′ ligation reaction, the SR RT Primer hybridized to the excess of 3′ SR Adaptor (that remained free after the 3′ ligation reaction) and transformed the single-stranded DNA adaptor into a double-stranded DNA molecule. This step is important to prevent adaptor-dimer formation, besides, dsDNAs are not substrates for ligation mediated by T4 RNA Ligase 1 and therefore do not ligate to the 5′ SR Adaptor in the subsequent ligation step. 5′ ends adapter was ligated to 5′ends of miRNAs, siRNA and piRNA. Then first strand cDNA was synthesized using M-MuLV Reverse Transcriptase. PCR amplification was performed using Long Amp Taq 2X Master Mix, SR Primer for illumina and index (X) primer. PCR products were purified on 8% polyacrylamide gel (100V, 80 min). DNA fragments corresponding to 140–160 bp were recovered and dissolved in 8 μL elution buffer. At last, library quality was assessed on the Agilent Bioanalyzer 2100 system using DNA High Sensitivity Chips. The library preparations were sequenced on an Illumina Hiseq 2500 platform and 50bp single-end reads were generated.

The sequencing quality of raw data of fastq format was assessed with FastQC and then low quality data were filtered using NGSQC. The clean reads with high quality were then aligned to the reference genome using Tophat2 with default parameters. The genome of human version of hg38 (UCSC) was used as the reference. Software of Cufflinks and Cuffmerge was used to assembly the transcripts. All the subsequent comparing analyses of lncRNA and mRNA were based on the results of the transcripts. The noval transcripts will be treated as noval lncRNA when its sequence length >200 and predicted to be a non-coding RNA. The differential expression analyses were performed using limma package and edgeR. Functional annotation and enrichment analyses were performed using KOBAS 3.0. The target gene of the lncRNA were predicted based on cis- and trans- pattern based on location on reference genome and sequence similarity.

To obtain circRNAs, the raw sequencing data were analyzed using FastQC and fastp software to obtain clean reads. After being aligned with the reference genome sequence using Tophat2 software, circRNAs were identified using the find circ and CIRCexplorer2 toolset. circRNA annotation was performed using BEDTools, a powerful tool for genome arithmetic. After normalizing the circRNA expression levels between samples using the calculated number of reads per kilobase per million mapped reads, the differences in circRNA expression levels among different samples were compared using the limma software package. Differentially expressed circRNAs were defined as |log2fold change| ≥ 1 and *p*-value < 0.05.

To obtain miRNA, Raw data of fastq format were firstly processed through bcl2fastq. In this step, clean data were obtained by removing reads containing ploy-N, with 5′adapter contaminants, without 3′adapter or the insert tag, containing ploy A or T or G or C and low quality reads. At the same time, Q20, Q30, and GC-content of the raw data were calculated. Then, chose a certain range of length from clean reads to do all the downstream analyses. The small RNA tags were mapped to reference sequence by Bowtie-1.1-1 without mismatch to analyze their expression and distribution on the reference. Known miRNA alignment Mapped small RNA tags were used to looking for known miRNA. miRBase22.0 was used as reference, bowtie-1.1.1 were used to obtain the potential miRNA and draw the secondary structures. The characteristics of hairpin structure of miRNA precursor can be used to predict novel miRNA. The library construction and transcriptome sequencing were completed by Beijing CapitalBio Technology.

### GO and KEGG Pathway Analysis

Gene ontology (GO) analysis describes genes and gene products in terms of cellular component, molecular function, and biological process. Pathway analysis is an effective method to predict the potential biological function of differentially expressed genes. This analysis was used to determine the main pathway for significant enrichment of differentially expressed RNAs. *P* value and error detection rate indicate the significance and biological pathway of enrichment in a differentially expressed RNAs list. (*P*-value < 0.05).

### Construction of Related ceRNA Regulation Network

The study was based on transcriptome sequencing technology, and DESeq based on negative binomial distribution was used to screen differentially expressed RNAs. Based on the principle of ceRNA, the regulatory networks of circRNA-ceRNA and lncRNA-ceRNA were constructed according to the regulation and regulated relationship among lncRNA, circRNA, miRNA, and mRNA. ceRNA was predicted through TargetScan software according to lncRNA-mRNA, circRNA-mRNA co-expression analysis, and then speculated the relevant miRNA. This analysis is based on the assumption that the increase of circRNA expression will competitively adsorb miRNA and thus reduce the expression of miRNA, and inhibition of target mRNA will be weakened, respectly. The Cytoscape software was used to visually display the regulatory network, in which the shape was used to distinguish the different types of RNAs, and the color was used to distinguish the up-regulation and down-regulation of RNAs expression.

### Statistical Analysis

All statistical data were analyzed by using SPSS 22.0 software (SPSS Inc., Chicago, IL, USA). lncRNA, circRNA, and mRNA expressed differentially between HT group and non-HT group were analyzed by using Student's t tests. Statistical significance was considered as *P* < 0.05.

## Results

### Differential Expression Profiles of mRNA, lncRNA, circRNA and miRNA Between HT Group and Non-HT Group Patients

**Figure 1:**
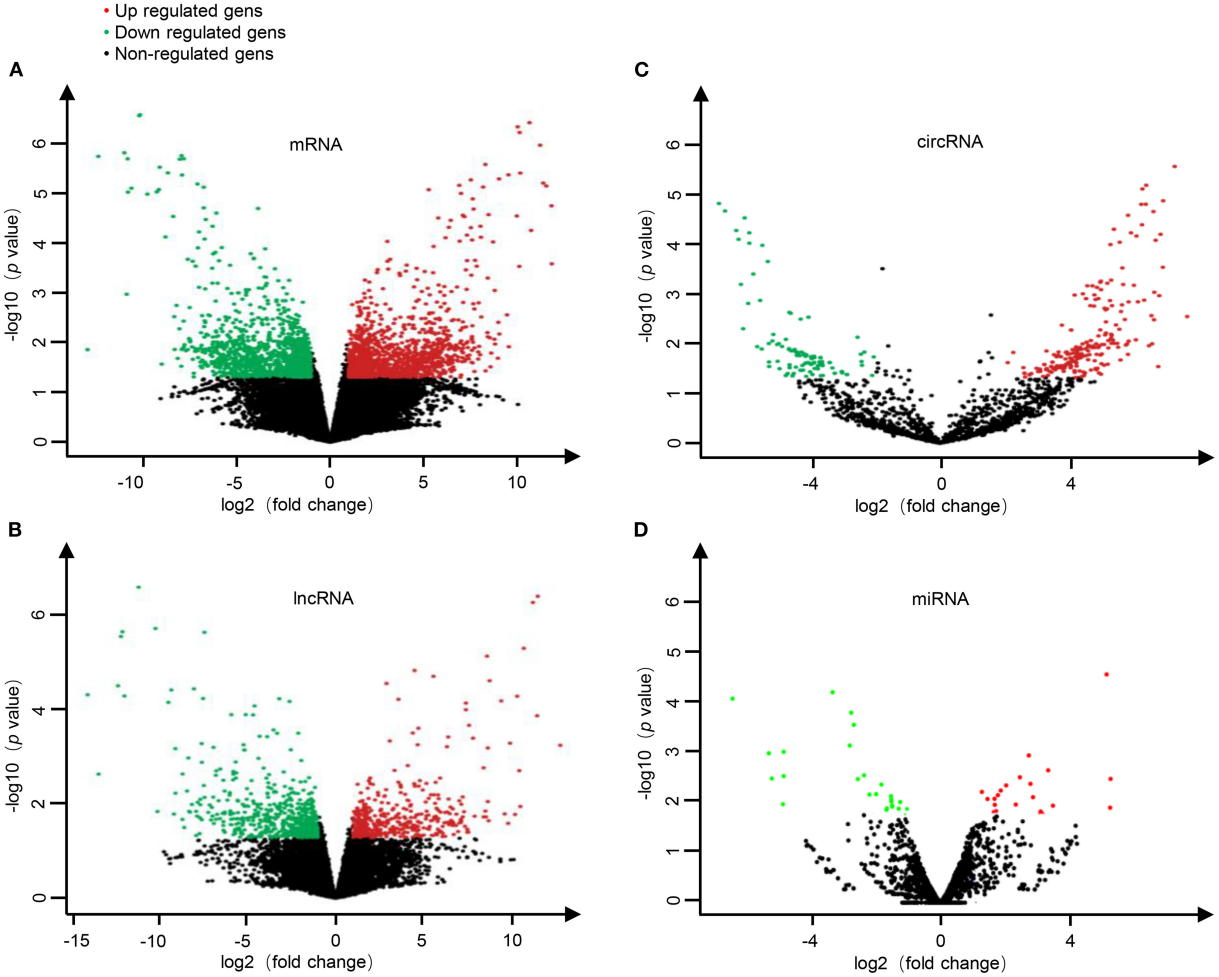
The volcano plots of differential mRNA **(A)**, lncRNA **(B)**, circRNA **(C)**, and miRNA **(D)** expression between HT group and non-HT group. Red dots represent up-regulated differential genes (fold change ≥2 and *p*-value < 0.05), green dots represent down-regulated differential genes (fold change ≤−2 and *p*-value < 0.05), and black dots represent genes with no significant differences.

**Figure 2:**
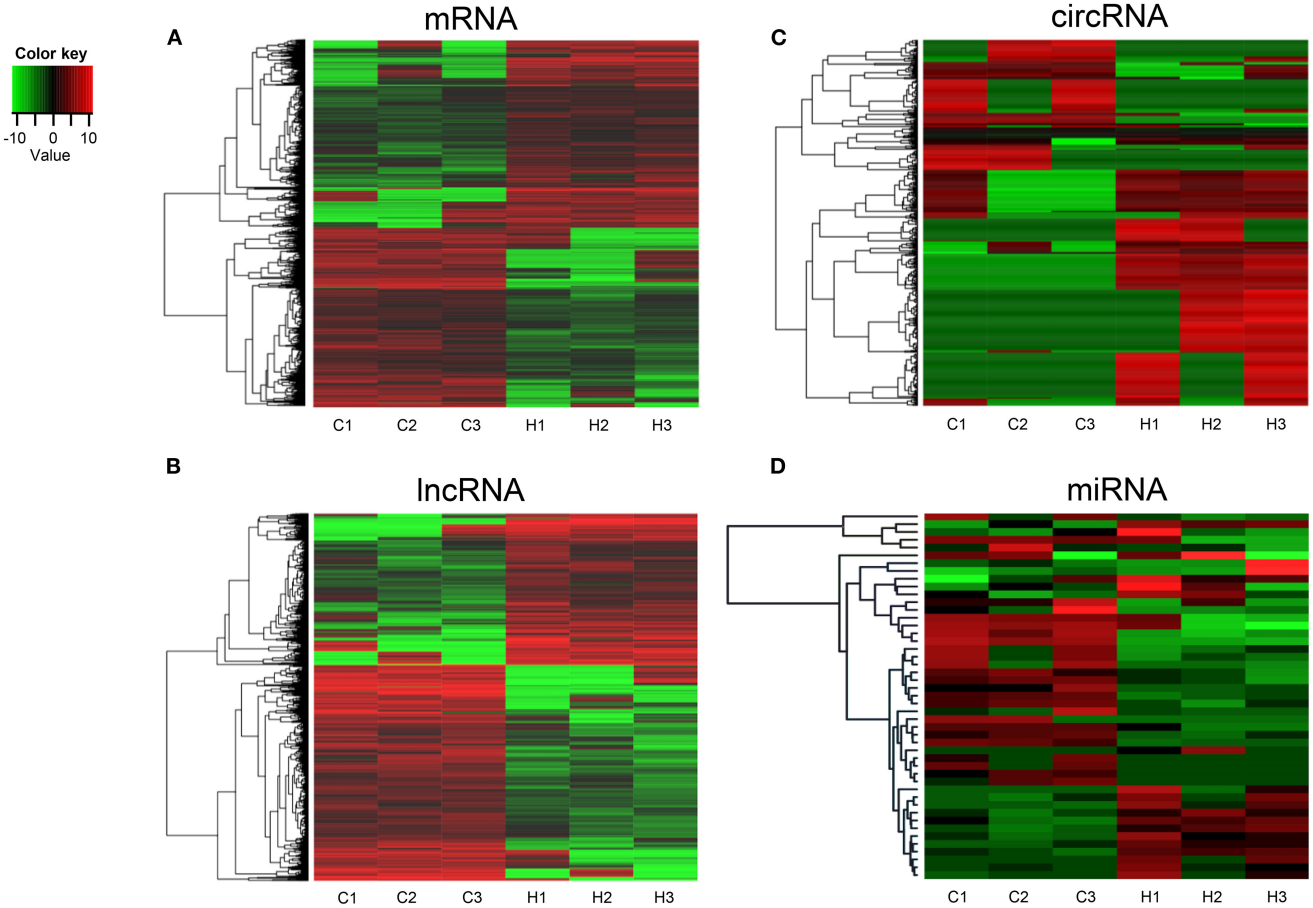
Hierarchical clustering of mRNA **(A)**, lncRNA **(B)**, circRNA **(C)**, and miRNA **(D)** in HT group and non-HT group. C1-C3: non-HT group; H1-H3: HT group. The red and the green shades indicate the expression above and below the relative expression, respectively, across all samples.

To investigate the expression levels of mRNA, lncRNA, circRNA and miRNA related to HT, peripheral blood mononuclear cells (PBMC) from 3 patients with HT and 3 patients without HT were analyzed by mRNA, lncRNA, circRNA and miRNA sequencing. Compared to the patients without HT, there were 1315 up-regulated mRNAs, 1260 down-regulated mRNAs; 433 up-regulated lncRNAs, 618 down-regulated lncRNAs; 301 up-regulated circRNAs,146 down-regulated circRNAs; 21 up-regulated miRNAs, 26 down-regulated miRNAs in patients with HT. Figure 1 is a volcanic diagram of mRNA (Figure 1A), lncRNA (Figure 1B), circRNA (Figure 1C), miRNA (Figure 1D) showing the up and down regulation between HT group and non-HT group. Figure 2 is a hierarchical clustering heat map showing the differentially expressed mRNA (Figure 2A), lncRNA (Figure 2B), circRNA (Figure 2C) and miRNA (Figure 2D) between HT group and non-HT group. We have selected the top10 of differentially expressed mRNAs, lncRNAs, circRNAs and miRNA. The detailed information was summarized in the following table (Tables 1–4).

**Table 1:** Top 10 of differently expressed mRNAs in HT and non-HT group (sorted by |log2FC|).

**Up-regulated mRNAs**				**Down-regulated mRNAs**			
**ID**	**Gene symbol**	**|log2FC|**	***P***	**ID**	**Gene symbol**	**|log2FC|**	***P***
ENST00000646036	IFNGR1	14.29458	9.79E-08	ENST00000573600	CTDNEP1	9.74614	1.05E-05
ENST00000357103	ADIPOR2	11.84416	0.000264	ENST00000394989	SNCA	9.24065	9.45E-06
ENST00000577035	GABARAP	11.82625	1.8E-05	ENST00000543581	HDC	9.13386	8.53E-06
ENST00000338010	ZNF385A	11.54945	7.2E-06	ENST00000515662	THUMPD3	9.08695	3.02E-06
ENST00000493678	PARK7	11.3872	6.3E-06	ENST00000344113	SYNE2	8.98145	0.027123
ENST00000485608	NRDC	11.22089	1.09E-06	ENST00000297438	OSGIN2	8.78100	7.58E-05
ENST00000541573	SOD2	10.73967	5.63E-05	ENST00000394909	ZNF16	8.65187	3.92E-06
ENST00000559161	PSTPIP1	10.65328	3.86E-07	ENST00000312037	RPS14	8.36715	2.94E-05
ENST00000305544	LAMB2	10.16102	3.96E-06	ENST00000526893	IGLL5	8.30961	0.003021
ENST00000443816	FN1	10.12476	6.08E-07	ENST00000588188	PRKAR1A	8.28907	0.01378

*FC, absolute fold change. “E” is the abbreviation of exponent (Index) in the scientific counting method*.

**Table 2:** Top 10 of differently expressed lncRNAs in HT and non-HT group.

**Up-regulated LncRNAs**				**Down-regulated LncRNAs**			
**ID**	**Gene symbol**	**|log2FC|**	***P***	**ID**	**Gene symbol**	**|log2FC|**	***P***
MERGE.10313.11	–	12.75585	0.000513	MERGE.14007.8	–	9.48446	6.1E-05
MERGE.33101.5	–	11.47681	3.18E-07	NONHSAT150565.1	–	9.31243	3.3E-05
MERGE.33002.11	–	11.43869	0.000118	MERGE.37406.11	–	9.17126	0.015436
MERGE.38119.13	–	11.20021	4.31E-07	MERGE.20852.5	–	9.08594	0.000602
MERGE.22405.3	–	10.6821	4.18E-06	MERGE.14007.15	–	9.08038	0.005487
MERGE.44550.2	–	10.48773	0.010613	MERGE.23352.2	–	8.68592	0.020124
MERGE.35461.3	–	10.42537	0.001802	MERGE.14003.9	–	8.67614	0.002119
MERGE.23722.3	–	10.30611	4.49E-05	MERGE.42338.1	–	8.58535	0.030032
MERGE.10313.11	–	12.75585	0.000513	MERGE.14003.31	–	8.42382	0.004927
MERGE.33101.5	–	11.47681	3.18E-07	MERGE.14003.51	–	8.34763	0.000957

**Table 3:** Top 10 of differently expressed circRNAs in HT and non-HT group.

**Up-regulated circRNAs**				**Down-regulated circRNAs**			
**ID**	**Gene symbol**	**|log2FC|**	***P***	**ID**	**Gene symbol**	**|log2FC|**	***P***
CBT15_circR_4799	AMD1	4.498915	1.22E-07	CBT15_circR_1945	DPP8	3.43764	1.77E-05
CBT15_circR_4963	HLA-C	3.847399	0.003077	CBT15_circR_4816	THEMIS	3.34245	2.49E-05
CBT15_circR_5157	KDM7A	3.652691	3.27E-06	CBT15_circR_2094	SMG1	3.16834	6.07E-05
CBT15_circR_3495	ZMYND8	3.473874	1.56E-05	CBT15_circR_2966	STK39	3.1322	9.07E-05
CBT15_circR_2054	MCTP2	3.468387	0.000325	CBT15_circR_723	CSGALNACT2	3.09571	0.00071
CBT15_circR_5215	CHST12	3.432919	7.22E-05	CBT15_circR_2013	SEC11A	3.06082	0.005363
CBT15_circR_278	RPS6KC1	3.412343	0.001186	CBT15_circR_3485	STK4	3.03802	3.42E-05
CBT15_circR_4914	IGF2R	3.396987	0.03013	CBT15_circR_5933	-	2.98242	0.001689
CBT15_circR_2885	ACTR3	3.356497	9.47E-05	CBT15_circR_2230	TERF2	2.96507	6.75E-05
CBT15_circR_5203	HDAC9	3.335417	0.001018	CBT15_circR_2266	ACSF3	2.96423	0.000108

**Table 4:** Top 10 of differently expressed miRNAs in HT and non-HT group.

**Up-regulated miRNAs**				**Down-regulated miRNAs**			
**ID**	**Gene symbol**	**|log2FC|**	***P***	**ID**	**Gene symbol**	**|log2FC|**	***P***
hsa-miR-548h-5p	MIR548H5	5.217028	0.015899	hsa-miR-544b	MIR544B	6.36173	0.001081
hsa-miR-208b-3p	MIR208B	5.201771	0.041621	hsa-miR-383-5p	MIR383	5.25119	0.006735
hsa-miR-1299	MIR1299	5.093736	0.00048	hsa-miR-4671-5p	MIR4671	5.1606	0.015671
hsa-miR-122-5p	MIR122	3.450962	0.038935	hsa-miR-99a-3p	MIR99A	4.81716	0.0371
hsa-miR-3675-5p	MIR3675	3.304885	0.011912	hsa-miR-155-3p	MIR155	4.79687	0.006418
hsa-miR-6509-3p	MIR6509	3.072799	0.047281	hsa-miR-5706	MIR5706	4.79656	0.014463
hsa-miR-548d-3p	MIR548D2	2.840033	0.029316	hsa-miR-708-3p	MIR708	3.3328	7.45E-07
hsa-miR-1295a	MIR1295A	2.762832	0.018769	hsa-miR-193b-5p	MIR193B	3.29713	0.000875
hsa-miR-4799-5p	MIR4799	2.711096	0.007246	hsa-miR-708-5p	MIR708	3.14699	2.95E-06
hsa-miR-548ar-3p	MIR548AR	2.438299	0.015047	hsa-miR-7974	MIR7974	2.77172	0.005195

### GO and KEGG Pathway Analysis of Differentially Expressed Genes

**Figure 3:**
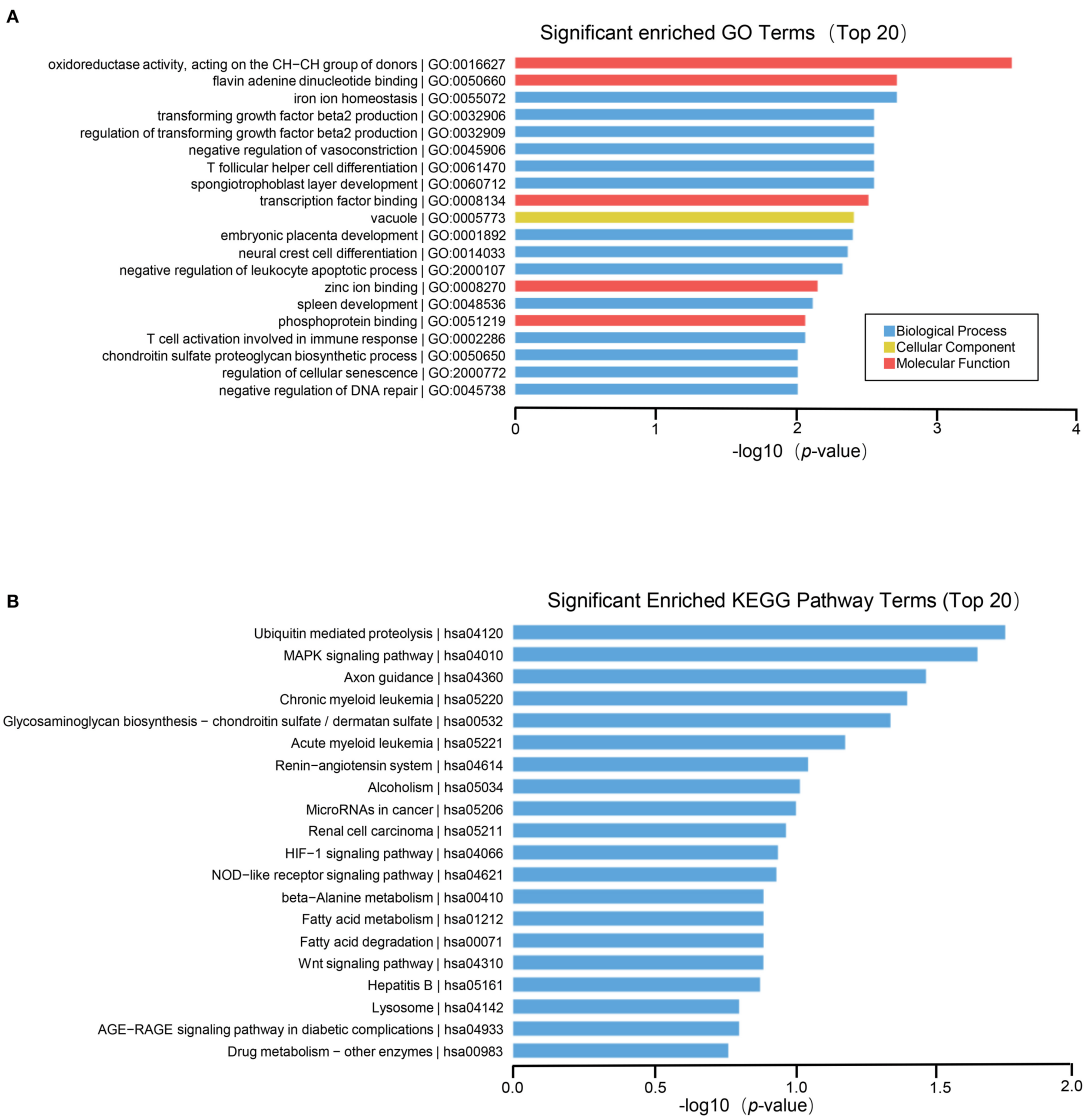
GO and KEGG pathway analysis on the results of circRNAs. **(A)** Gene ontology analysis. Top 20 GO terms for the difference in circRNA between HT group and non-HT group. **(B)** KEGG pathway analysis. Top 20 pathways for the difference in circRNA between HT group and non-HT group.

To further explore the role of differentially expressed genes in HT, we annotated the function of differentially expressed genes by using GO and KEGG path analysis on the results of mRNA, lncRNA, circRNA and miRNA transcriptome sequencing analysis. The biological processes of differentially expressed circRNA derived genes include iron ion homeostasis, transforming growth factor beta2 production, regulation of transforming growth factor beta2 production, negative regulation of vasoconstriction, T follicular helper cell differentiation, spongiotrophoblast layer development, and so on; The cell components involved include vacuole, golgi trans cisterna, endosome, nuclear inner membrane, etc. The molecular functions involved include oxidoreductase activity, acting on the CH–CH group of donors, flavin adenine dinucleotide binding, transcription factor binding, zinc ion binding, etc. (Figure 3A). In circRNA, the pathways with high enrichment in the KEGG pathway are ubiquitin-mediated proteolysis, MAPK signaling pathway, axon guidance, glycosaminoglycan biosynthesis-chondroitin sulfate/dermatan sulfate, HIF-1 signal pathway, NOD-like receptor signaling pathway, beta–alanine metabolism, Wnt signaling pathway, and so on (Figure 3B).

**Figure 4:**
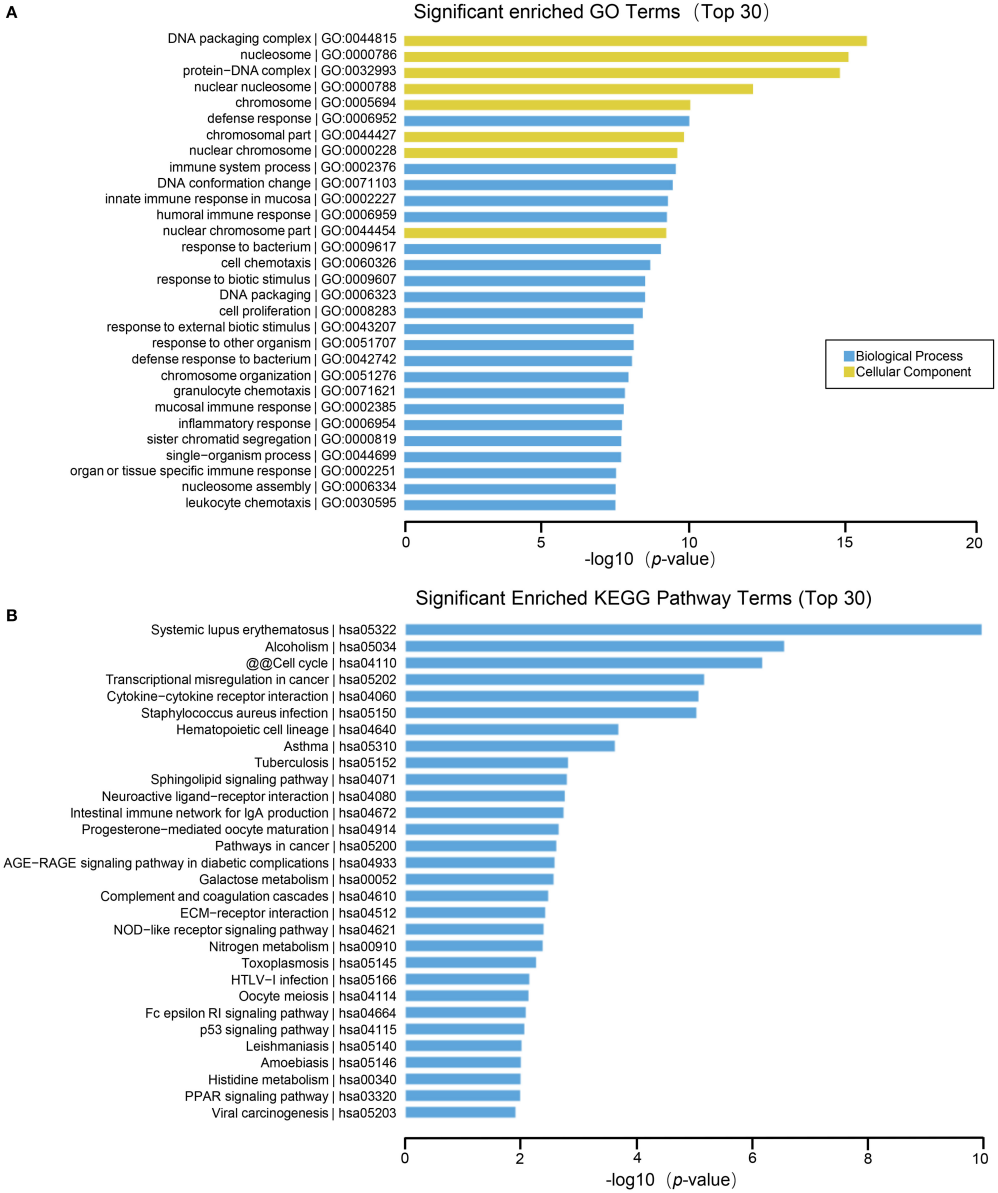
GO and KEGG pathway analysis on the results of lncRNAs. **(A)** Gene ontology analysis. Top 30 GO terms for the difference in lncRNA between HT group and non-HT group. **(B)** KEGG pathway analysis. Top 30 pathways for the difference in lncRNA between HT group and non-HT group.

The results of GO function analysis showed that the main biological processes involved in differentially expressed lncRNAs include defense response, immune system process, DNA conformational change, mucosal innate immune response in mucosa, humoral immune response, cell chemotaxis, cell proliferation, etc. The cell components involved include DNA packaging complex, nucleosome, protein-DNA complex, nuclear nucleosomes, chromosome, nuclear chromosome, nuclear chromatin, plasma membrane, etc. The molecular functions involved include: anaphylatoxin receptor activity, molecular transducer activity, receptor activity, protein heterodimerization activity, immunoglobulin binding, signal transducer activity, glycosaminoglycan binding, protein binding, oxygen transporter activity, signaling receptor activity, etc. (Figure 4A). In lncRNA, the pathways with high enrichment in the KEGG pathway include systemic lupus erythematosus, alcoholism, cell cycle, transcriptional misregulation in cancer, cytokine-cytokine receptor interactions, staphylococcus aureus infection, hematopoietic cell lineage, asthma, tuberculosis, sphingolipid signaling pathway, neuroactive ligand-receptor interaction, intestinal immune network for IgA production, etc. (Figure 4B).

**Figure 5:**
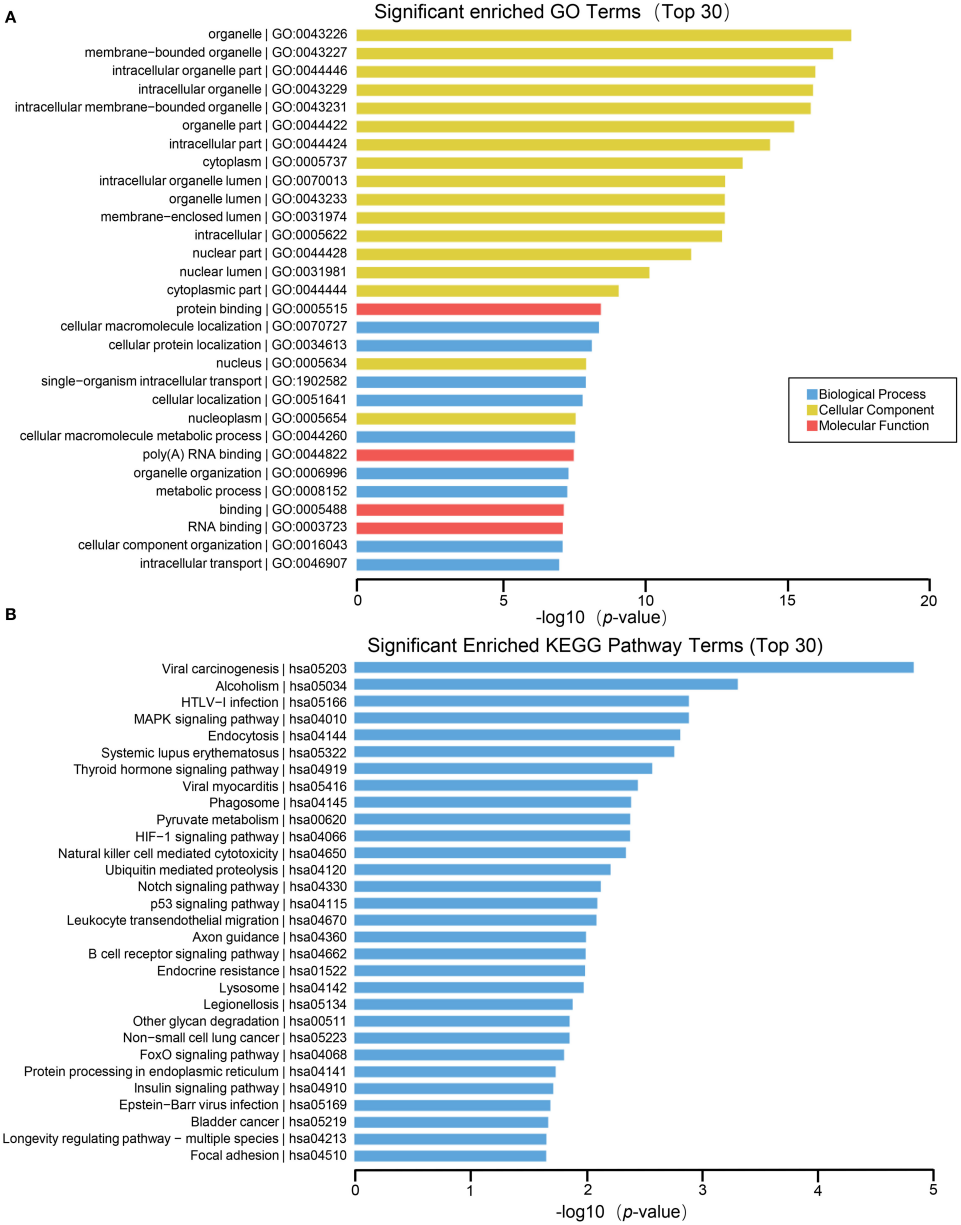
GO and KEGG pathway analysis on the results of mRNAs. **(A)** Gene ontology analysis. Top 30 GO terms for the difference in mRNA between HT group and non-HT group. **(B)** KEGG pathway analysis. Top 30 pathways for the difference in mRNA between HT group and non-HT group.

Functional enrichment analysis of differentially expressed mRNAs suggested that the main biological processes involved include cellular macromolecule localization, cellular protein localization, single–organism intracellular transport, cellular localization, cellular macromolecule metabolic process, organelle organization, metabolic process, cellular component organization, intracellular transport, etc. The molecular functions involved include membrane–bounded organelle, intracellular organelle part, intracellular organelle, intracellular membrane–bounded organelle, organelle part, intracellular part, cytoplasm, intracellular organelle lumen, organelle lumen, membrane–enclosed lumen, intracellular, nuclear part, nuclear lumen, cytoplasmic part, nucleus, nucleoplasmn, etc. The molecular functions involved include: protein binding, poly A RNA binding, RNA binding, etc. (Figure 5A). Many pathways are enriched in the KEGG analysis result such as MAPK signaling pathway, endocytosisn, thyroid hormone signaling pathway, phagosomen, pyruvate metabolism, HIF−1 signaling pathway, patural killer cell mediated cytotoxicity, ubiquitin mediated proteolysis, Notch signaling pathway, signaling pathway, leukocyte transendothelial migration, axon guidance, B cell receptor signaling pathway, Endocrine resistance, FoxO signaling pathway, Protein processing in endoplasmic reticulum, Insulin signaling pathway, etc. (Figure 5B).

**Figure 6:**
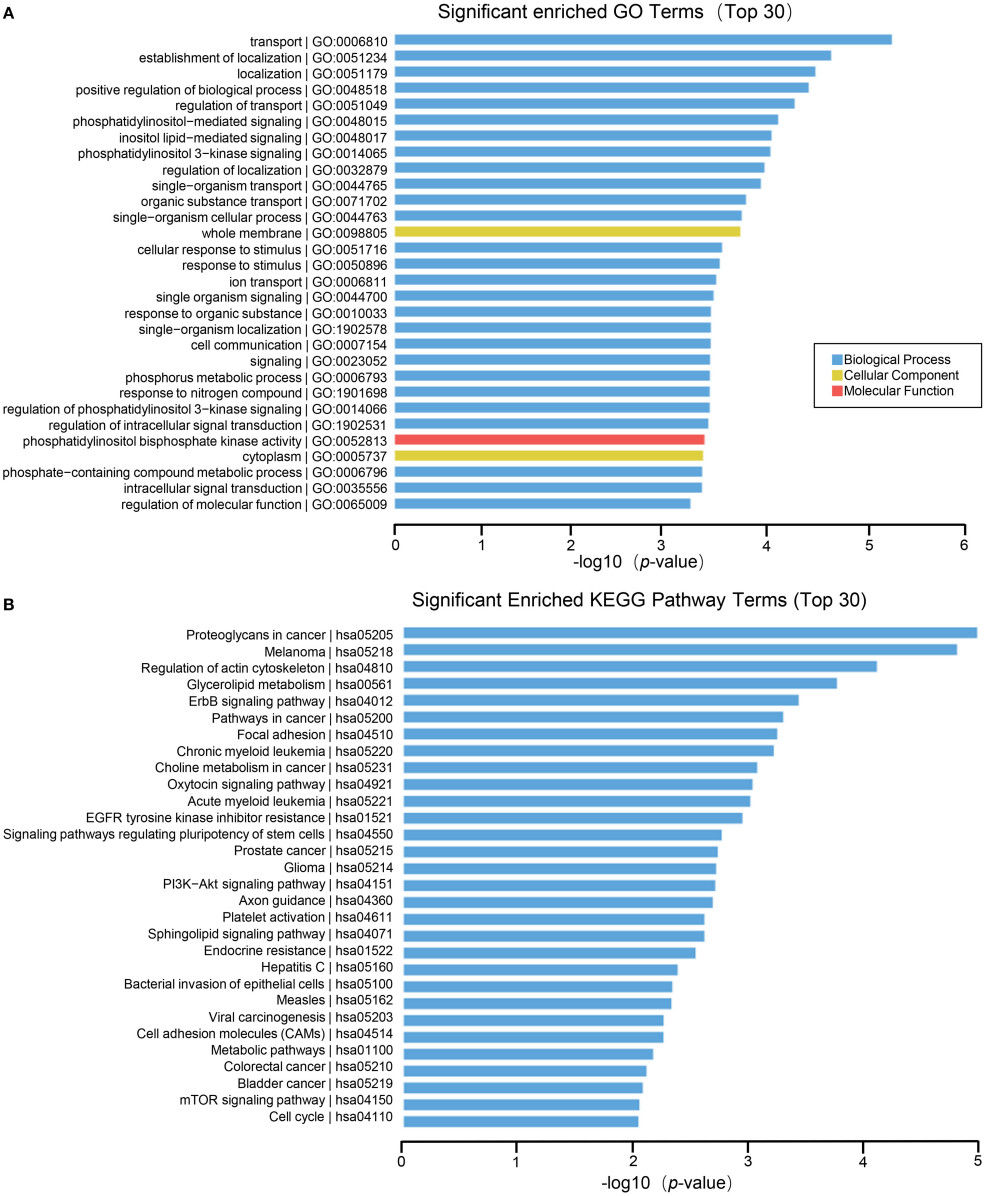
GO and KEGG pathway analysis on the results of miRNAs. **(A)** Gene ontology analysis. Top 30 GO terms for the difference in miRNA between HT group and non-HT group. **(B)** KEGG pathway analysis. Top 30 pathways for the difference in miRNA between HT group and non-HT group.

For miRNA, the biological processes of differentially expressed genes contain transport, establishment of localization, positive regulation of biological process, phosphatidylinositol–mediated signaling, inositol lipid–mediated signaling, phosphatidylinositol 3–kinase signaling, etc. The cell components involved whole membrane and cytoplasm. The molecular function include phosphatidylinositol bisphosphate kinase activity (Figure 6A). Furthermore, the results of KEGG enrichment analysis comprise proteoglycans in cancer, regulation of actin cytoskeleton, glycerolipid metabolism, ErbB signaling pathway, signaling pathways regulating pluripotency of stem cells, PI3K–Akt signaling pathway and so on (Figure 6B).

## Analysis of Differential Gene Co-Expression Network

### Results of Correlation Analysis Between lncRNA and miRNA

**Figure 7:**
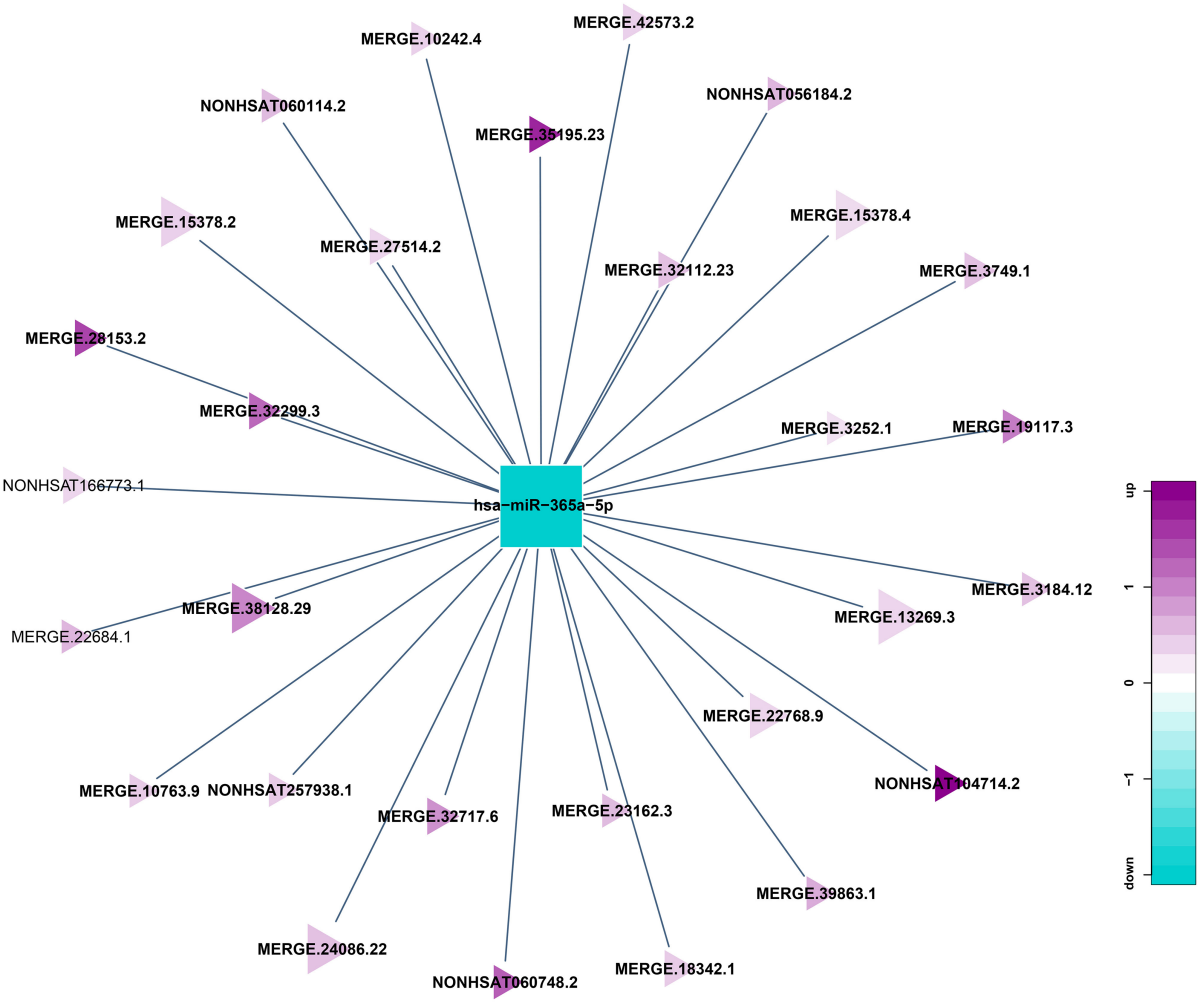
Correlation analysis diagram of hsa-miR-365a-5p-lncRNA (Squares: miRNA; Triangle: lncRNA; Blue: down-regulation; Purple: up-regulation).

miRNA can be combined with lncRNA to affect the function of lncRNA, and lncRNA can also adsorb miRNA to affect the function of it. Because of the interaction between lncRNA and miRNA, miRNA binding site analysis of differentially expressed lncRNA might enhance our comprehension of lncRNA. In this research, miRanda software is used to predict the targeted lncRNA of miRNA in the sample, and then Cytoscape software is used to build lncRNA and miRNA networks. A total of 261 lncRNA-miRNA relationship pairs were obtained, of which 175 miRNAs were down-regulated and lncRNAs were up-regulated, and 86 miRNAs were up-regulated and lncRNA were down-regulated. The largest network team is hsa-miR-365a-5p, which crosstalk with 29 lncRNAs (Figure 7), this may indicate 29 lncRNAs could modulate the cellular functions by regulating hsa-miR-365a-5p.

### Results of Correlation Analysis Between circRNA and miRNA

**Figure 8:**
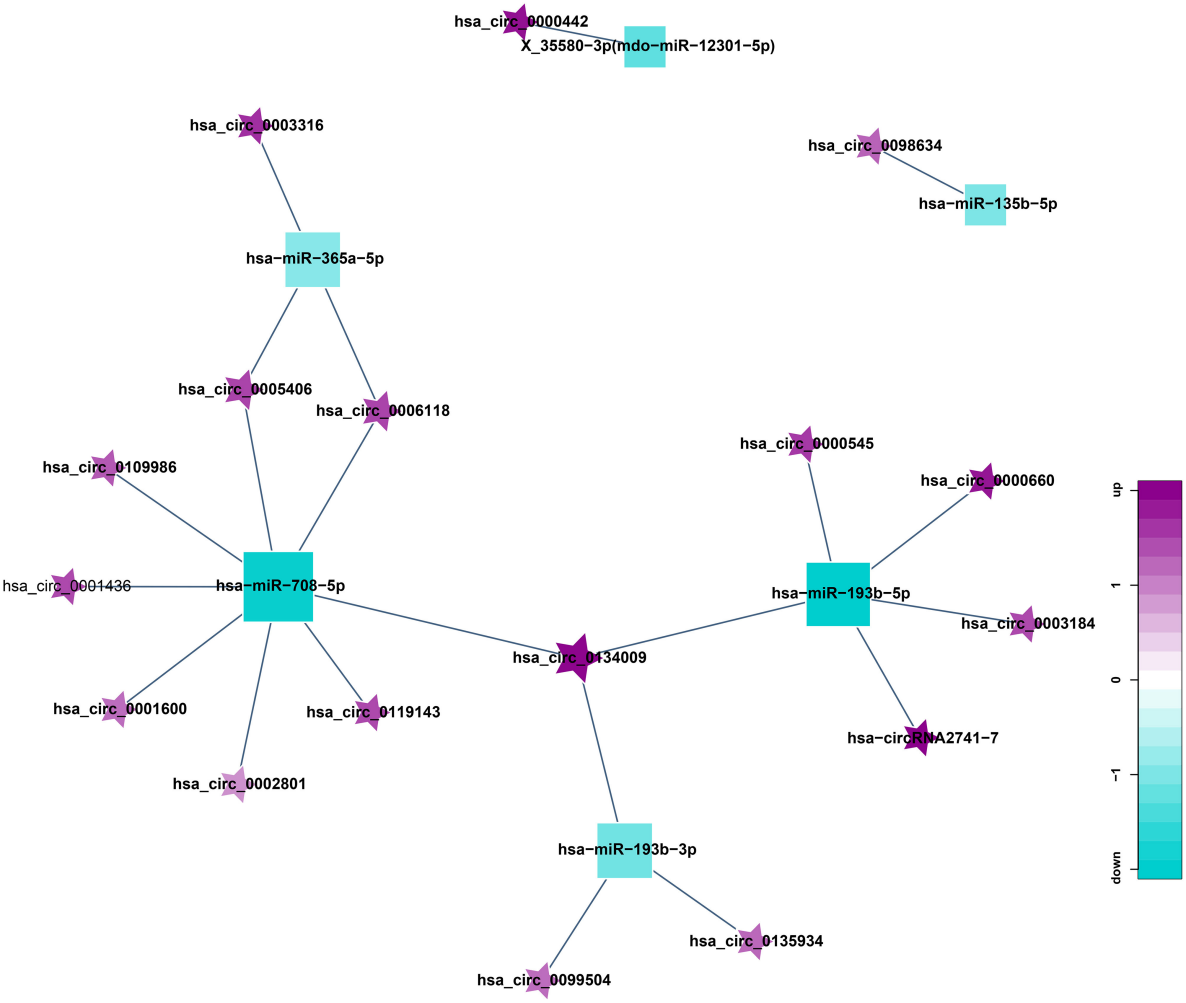
Correlation analysis diagram of circRNA-miRNA (Square: miRNA; Pentagram: circRNA; Blue: down-regulation; Purple: up-regulation).

circRNA can suppress the function of miRNA by combining with miRNA. Therefore, miRNA binding sites analysis of the differentially expressed circRNA is helpful to further study the function of circRNA. According to the predetermined results of miRNA binding sites, a total of 21 circRNA-miRNA relationship pairs were obtained from the regulatory network of circRNA-miRNA (Figure 8). Among them, 8 circRNAs could established a relationship with hsa-miR-708-5p. In addition, hsa_circ_0098634 and hsa-miR-135b-5p, hsa_circ_0000442, and X_35580-3p (mdo-miR-12301-5p) only establish a relationship with each other, and these miRNAs and circRNAs may play a key role in HT.

### Construction of ceRNA Network Mediated by Differentially Expressed circRNA

**Figure 9:**
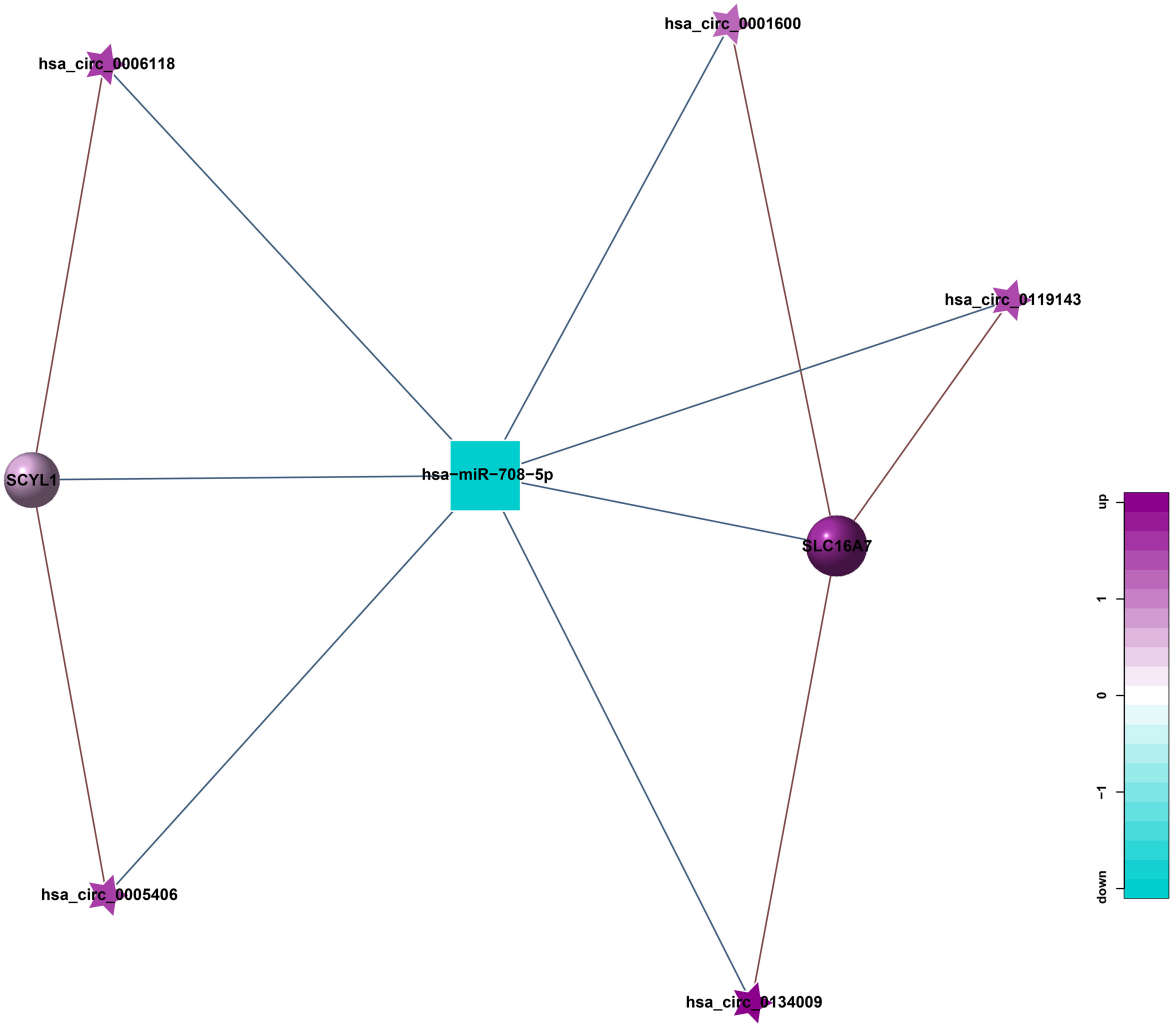
circRNA-miRNA-mRNA regulatory network related to HT (Pentagram: circRNA; Square: miRNA; Circle: mRNA; Blue: down-regulation; Purple: up-regulation).

ceRNA is a hot topic in recent years. It refers to a kind of non-coding RNA that can bind to miRNA response element, inhibit the formation of miRNA, thereby increasing the expression of the corresponding mRNA, and finally achieving the regulation of gene expression. ceRNA mainly includes lncRNA, circRNA, and pseudogenes. An increasing amount of evidence shows that some circRNA can be used as ceRNA to affect the distribution of miRNA in target genes. Therefore, we constructed a circRNA-mediated ceRNA network differentially expressed in HT (Figure 9). We screened 5 circRNAs from the differentially expressed circRNAs, namely hsa_circ_0001600, hsa_circ_0006118, hsa_circ_0119143, hsa_circ_0134009, and hsa_circ_0005406. They can act as ceRNA to compete with hsa-miR-708-5p and regulate the function of SCYL1, SLC16A7 in the network. This be associated with HT.

### Construction of ceRNA Network Mediated by Differentially Expressed lncRNA

**Figure 10:**
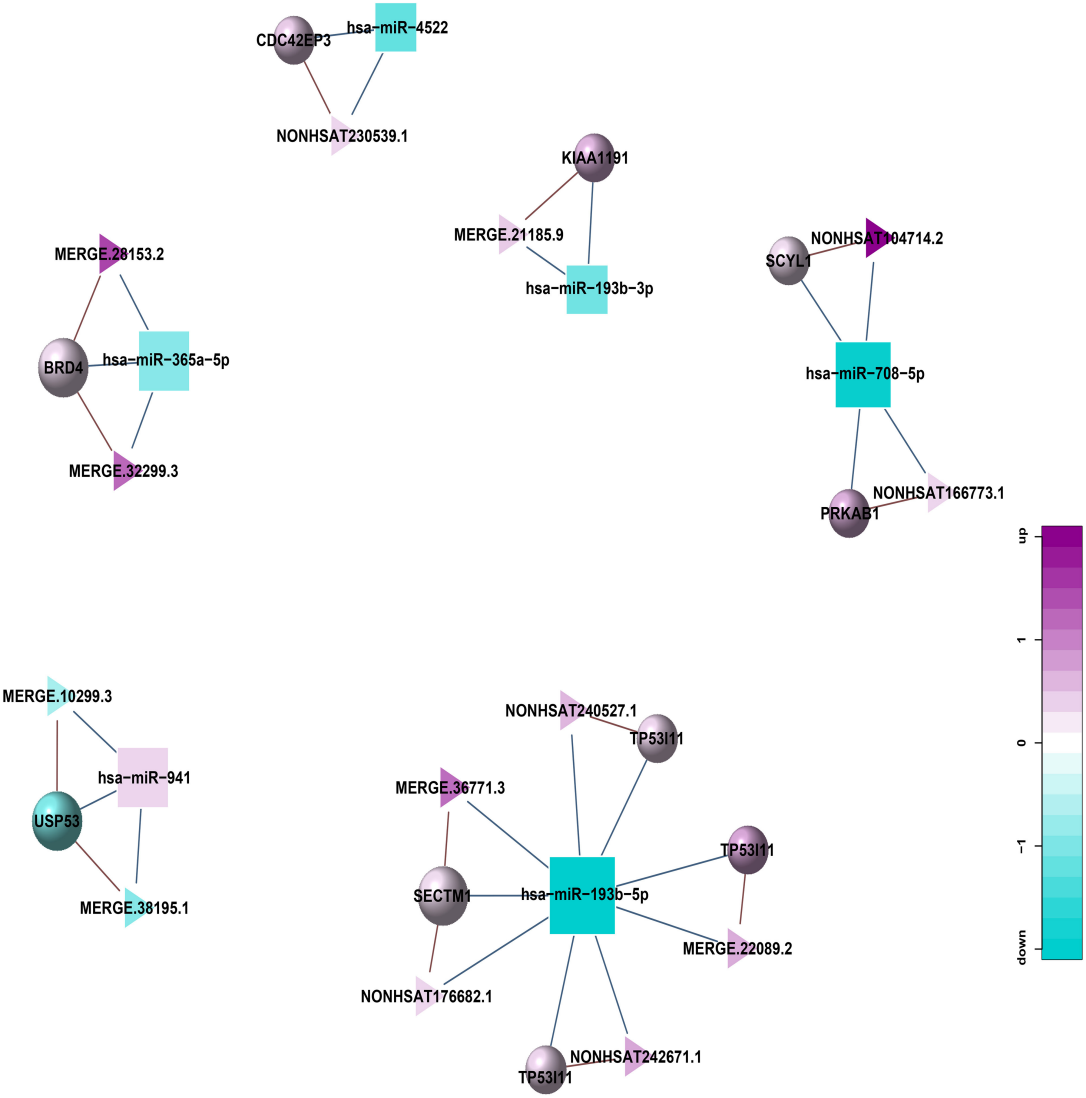
lncRNA-miRNA-mRNA regulatory network related to HT (Triangle: lncRNA; Square: miRNA; Circle: mRNA; Blue: down-regulation; Purple: up-regulation).

In addition, to further explore the role of lncRNA in the pathogenesis of HT, we also constructed a lncRNA-mediated ceRNA network by differentially expressed genes in HT (Figure 10) and used Arraystar data, TargetScan, and miRanda to predict the interaction among lncRNA, mRNA, and miRNA. Compared with the traditional sequencing analysis only for a single type of RNA, the application of ceRNA theory can show the relationship between regulated and be regulated of RNA, and to obtain more scientific and rigorous research results. By establishing the above regulatory network, we screened 13 lncRNAs from the differentially expressed lncRNAs, which can be used as ceRNA to participate in the regulation of the function of 10 mRNAs in the network by competing with 6 miRNAs in the network, thus might associate with HT.

## Discussion

HT is a serious complication of acute ischemic stroke, which is independently related to neurodegeneration and functional deterioration. It is demonstrated that the mechanism of HT after ischemic stroke structurally occurs when peripheral blood infiltrates into the brain through the damaged blood-brain barrier (BBB) (Spronk et al., 2021). Specific pathophysiological mechanisms may involve cascade reactions such as reperfusion injury, oxidative stress, leukocyte infiltration, vascular activation, and imbalance of extracellular proteolysis, which destroy the integrity of the basal layer and endothelial cells and ultimately may trigger HT (Krishnamoorthy et al., 2021). In addition, some scholars believe that neuroinflammation, vascular endothelial growth factor, reactive oxygen species, and matrix metalloproteinase-2 are also involved in the destruction of the BBB and the occurrence of HT after cerebral infarction and ischemia-reperfusion (Spronk et al., 2021). However, the potential mechanism and biomarkers of HT are still worthy for further exploration. Therefore, we try to describe the epigenetic characteristics of the disease and reveal the complex interactions between its transcripts by analyzing the differentially expressed mRNA, lncRNA, circRNA and miRNA between HT and non-HT group, and try to provide useful information for further understanding its mechanism and exploring potential targets for treatment and prognosis.

Using the sequencing technique, we showed for the first time the differential expression profile between patients with HT and patients without HT after acute ischemic stroke. Compared with the non-HT group, 1051 lncRNAs, 2575 mRNAs, 447 circRNAs and 47miRNAs were differentially expressed in the HT group. Moreover, we identified the potential function of these differentially expressed RNAs by GO and KEGG pathway analysis. At the same time, we draw the ceRNA regulation network diagram of circRNA-miRNA-mRNA and lncRNA-miRNA-mRNA by combining transcriptome data. The regulatory network map of ceRNA plays a guiding and helpful role in discovering and screening circRNA and ceRNA networks which are at the core of network regulation and can further narrow the scope of research. It provides a theoretical basis for further study of circRNA, lncRNA function, and other transcriptome information related to HT.

Transcriptome sequencing provides an opportunity to detect all transcripts in specific tissues and is widely used in neurological diseases. In this study, transcriptome sequencing was used to compare the expression profiles of lncRNA, circRNA, mRNA and miRNA between HT and non-HT patients. It was found that there were 1051 lncRNAs (433 up-regulated lncRNAs, 618 down-regulated lncRNAs), 2575 mRNAs (1315 up-regulated mRNAs, 1260 down-regulated mRNAs), 447 circRNAs (301 up-regulated circRNAs, 146 down-regulated circRNAs) and 47 miRNAs (21 up-regulated miRNAs, 26 down-regulated miRNAs) between HT and non-HT patients. It provides a possible opportunity for the early diagnosis of HT by using these differentially expressed genes and ribonucleic acid.

Transcripts that exceed 200 nucleotides and most of them could not have coding ability are usually defined as lncRNAs. It has been proved that lncRNA is involved in epigenetic regulation, transcription, translation, RNA metabolism, autophagy, apoptosis, and so on (Deng et al., 2018; Qian et al., 2019; Zhang and Wang, 2019). Although only a small number of identified lncRNAs have been deeply studied, they play an important role in various physiological and pathological processes such as cell differentiation, tumorigenesis, metastasis, immune response, aging, and so on(Peng and Li, 2019). Recent studies have also recognized that lncRNA is closely related to the BBB permeability. In a study on intracerebral hemorrhage, it was found that the up-regulation of LncRNA SNHG3 can increase the permeability of BBB by activating TWEAK12/Fn14/STAT3 signal pathway (Ma et al., 2018; Zhang et al., 2019). At present, the research shows that circRNA is another important part of the regulation of ncRNAs. circRNA is particularly abundant in the human brain, peripheral blood, and exocrine bodies, and their ability of passing through the BBB makes them perfect candidates for potential markers of the diagnosis of central nervous system diseases (Hanan et al., 2017). Many studies have reported that circRNA can affect cell proliferation, autophagy, and differentiation (Jeck and Sharpless, 2014; Fu and Sun, 2021). In addition, there are more and more reports about the transcriptional spectrum of circRNA in different models of central nervous system diseases, such as stroke, meningitis, Parkinson's disease, and Alzheimer's disease (Zhang et al., 2017b; Kumar et al., 2018; Liu et al., 2019).

We analyzed the GO and KEGG pathways to obtain detailed information about the biological functions and potential mechanisms of these differentially expressed mRNA, lncRNA, circRNA and miRNA. Pathway analysis showed that ubiquitin-mediated proteolysis, MAPK signal pathway, axon guidance, HIF-1 signal pathway, NOD-like receptor signal pathway, beta-alanine metabolism, Wnt signal pathway, sphingolipid signal pathway, neuroactive ligand-receptor interaction, and intestinal immune network used in IgA production play a vital role in HT. Terms such as iron homeostasis, defense response, immune system process, DNA conformational change, production of transforming growth factor beta2, and oxidoreductase activity are enriched in the gene list, suggesting a potential correlation with HT. This is consistent with the known research results. It is well known that the MAPK signal pathway is related to inflammation and apoptosis. Shi et al. demonstrated that MAPK signal pathway is the most abundant pathway in neutrophils of middle cerebral artery occlusion (MCAO) rats after tPA thrombolysis. MAPK family member genes are highly expressed in neutrophils of MCAO rats treated with tPA, suggesting that the MAPK pathway may mediate the activation of immune cells after tPA injection, which leads to the occurrence of HT after stroke (Shi et al., 2021). The Wnt signal pathway is involved in the formation of BBB, which is helpful to stimulate neurogenesis, consolidate blood-brain structure, and restore cognitive brain function after central nervous system injury (Zlokovic, 2011; Lambert et al., 2016; Wei et al., 2018). Chang et al. pointed out that the GPR124-Wnt signal axis is very important for the BBB and vascular integrity (Chang et al., 2017). In addition, some studies have found that glycogen synthesizer kinase 3 (GSK-3) beta inhibitor TWS119 may suppress rtPA-induced HT and decrease BBB damage by activating Wnt/β-catenin signaling pathway in rats with acute ischemic stroke (Wang et al., 2016, 2017). Hypoxia inducible factor 1a (HIF-1a) is a transcription factor that plays an important role in hypoxia perception and adaptation. It can also be activated by reactive oxygen species-mediated by cytokines, such as tumor necrosis factor (TNF) (Chen et al., 2018). HIF-1 and its downstream genes including MMP-2/9 and vascular endothelial growth factor (VEGF) are involved in the development of HT. The inhibition of HIF-1a and its downstream genes can reduce HT in cerebral infarction and improve neurological dysfunction after focal cerebral ischemia (Wu X. et al., 2018).

Through the construction of the co-expression network, we obtained a total of 261 lncRNA-miRNA relationship pairs, with the largest number of lncRNAs establishing relationships with hsa-miR-365a-5p, up to 29; 21 circRNA-miRNA relationship pairs were obtained, of which 8 circRNAs were established with hsa-miR-708-5p. 5 circRNAs, 13 lncRNAs were screened out to be used as ceRNA to compete with miRNA in the co-expression network, which can be associated with HT by regulating the function of mRNA in the network.

## Conclusion

There are still some limitations in this study. First of all, it is a small sample study, and a few patients with severe symptomatic intracranial hemorrhage and subarachnoid hemorrhage who cannot bear MR imaging are not included in this study, which may lead to case selection bias. In addition, this research did not carry out quantitative real-time PCR to further verify the results of transcriptome sequencing, which is what we will do in the next step. Finally, although we have carried out some basic bioinformatics analysis and constructed an HT co-expression network, further experiments (*in vivo* or *in vitro*) are needed to verify the function of these RNAs.

In short, the above results of this study suggest that the mutual regulation of mRNA, lncRNA, circRNA and miRNA may play a certain role in the occurrence of HT. With the development of gene sequencing technology, more and more ncRNAs related to HT will be identified in the future, and the interaction between them will be clarified, which will help to explore the epigenetics and related molecular details of HT after acute ischemic stroke, and provide new theoretical and data support for early diagnosis, appropriate treatment and improving the prognosis of patients with HT.

## Data Availability Statement

The datasets presented in this study can be found in online repositories. The name of the repository and accession number can be found below: NCBI Gene Expression Omnibus, GSE199435.

## Ethics Statement

The studies involving human participants were reviewed and approved by the Ethics Committee of the Affiliated Hospital of Jining Medical University. The patients/participants provided their written informed consent to participate in this study. Written informed consent was obtained from the individual(s) for the publication of any potentially identifiable images or data included in this article.

## Author Contributions

RH designed and performed experiments, prepared figures, analyzed data, and wrote, edited, and proofread the manuscript. PZ analyzed and interpreted data, prepared figures, and wrote, edited, and proofread the manuscript. HL and YC performed experiments. YH and QG assisted with patient studies. DL conceptualized the project and wrote, edited, and proofread the manuscript. AZ conceptualized and directed the overall project. All authors contributed to the article and approved the submitted version.

## Funding

This work was supported by grants from the National Natural Science Foundation of China (81901228).

## Conflict of Interest

The authors declare that the research was conducted in the absence of any commercial or financial relationships that could be construed as a potential conflict of interest.

## Publisher's Note

All claims expressed in this article are solely those of the authors and do not necessarily represent those of their affiliated organizations, or those of the publisher, the editors and the reviewers. Any product that may be evaluated in this article, or claim that may be made by its manufacturer, is not guaranteed or endorsed by the publisher.
